# Labour management guidelines for a Tanzanian referral hospital: The participatory development process and birth attendants’ perceptions

**DOI:** 10.1186/s12884-017-1360-2

**Published:** 2017-06-07

**Authors:** Nanna Maaløe, Natasha Housseine, Jos van Roosmalen, Ib Christian Bygbjerg, Britt Pinkowski Tersbøl, Rashid Saleh Khamis, Birgitte Bruun Nielsen, Tarek Meguid

**Affiliations:** 1 Global Health Section, Department of Public Health, University of Copenhagen, Øster Farimagsgade 5, Building 9, 1353 Copenhagen K, Denmark; 2 Department of Obstetrics and Gynaecology, Mnazi Mmoja Hospital, Zanzibar, Tanzania; 3 Julius Center for Health Sciences and Primary Care, University Medical Center Utrecht, Universiteitsweg 100, 3584 CG Utrecht, the Netherlands; 4 Athena Institute, VU University of Amsterdam, De Boelelaan 1105, 1081 HV Amsterdam, the Netherlands; 5 Department of Obstetrics, Rigshospitalet, Copenhagen University Hospital, Blegdamsvej 9, 2100 København Ø, Denmark; 6 School of Health & Medical Sciences, State University of Zanzibar, P.O.Box:146, Zanzibar, Tanzania

**Keywords:** Guidelines, Labour, Partograph, Quality of care, Tanzania, PartoMa

## Abstract

**Background:**

While international guidelines for intrapartum care appear to have increased rapidly since 2000, literature suggests that it has only in few instances been matched with reviews of local modifications, use, and impact at the targeted low resource facilities. At a Tanzanian referral hospital, this paper describes the development process of locally achievable, partograph-associated, and peer-reviewed labour management guidelines, and it presents an assessment of professional birth attendants’ perceptions.

**Methods:**

Part 1: Modification of evidence-based international guidelines through repeated evaluation cycles by local staff and seven external specialists in midwifery/obstetrics. Part 2: Questionnaire evaluation 12 months post-implementation of perceptions and use among professional birth attendants.

**Results:**

Part 1: After the development process, including three rounds of evaluation by staff and two external peer-review cycles, there were no major concerns with the guidelines internally nor externally. Thereby, international recommendations were condensed to the eight-paged ‘PartoMa guidelines ©’. This pocket booklet includes routine assessments, supportive care, and management of common abnormalities in foetal heart rate, labour progress, and maternal condition. It uses colour codes indicating urgency. Compared to international guidelines, reductions were made in frequency of assessments, information load, and ambiguity. Part 2: Response rate of 84% (*n* = 84). The majority of staff (93%) agreed that the guidelines helped to improve care. They found the guidelines achievable (89%), and the graphics worked well (90%). Doctors more often than nurse-midwives (89% versus 74%) responded to use the guidelines daily.

**Conclusions:**

The PartoMa guidelines ensure readily available, locally achievable, and acceptable support for intrapartum surveillance, triage, and management. This is a crucial example of adapting evidence-based international recommendations to local reality.

**Trial registration:**

This paper describes the intervention of the PartoMa trial, which is registered on ClinicalTrials.org (NCT02318420, 4th November 2014).

**Electronic supplementary material:**

The online version of this article (doi:10.1186/s12884-017-1360-2) contains supplementary material, which is available to authorized users.

## Background

An estimated 303,000 maternal deaths occur worldwide annually with the highest risk at the time of birth, and 3 million babies die as intrapartum stillbirths or early neonatal deaths [1–3]. Global efforts have focused on increasing facility births. However, these have not been matched with actual skilled care and this is an urgent post-2015 priority [4–8].

While promising interventions have been launched concerning e.g. postpartum bleeding and neonatal resuscitation, evidence on effective intrapartum interventions are limited [6, 9–11]. Meanwhile, many complications needing emergency postpartum/neonatal management could be prevented by basic and timely labour care [6, 12]. The World Health Organization’s (WHOs) partograph is generally perceived to be central for improving intrapartum care [13–15]. However, as shown in the WHO’s Asian multi-centre trial of 35,484 deliveries, partograph use should be coupled with realistic and simple management guidelines to achieve effect [16]. Moreover, guidance of health providers in best possible intrapartum care appears crucial [5, 8, 17]. Yet, a gap exists between international evidence-based guidelines for low resource settings and what is achievable and applicable locally [6, 18].

**Fig. 1:**
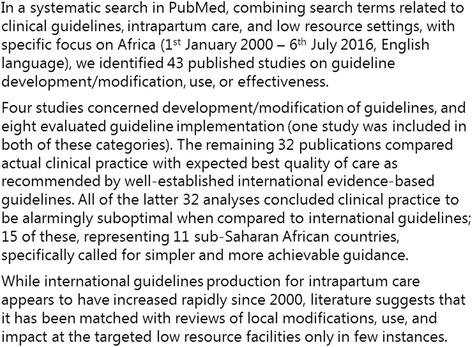
Systematic literature search: Labour and delivery guidelines for African low-income settings. A more detailed search description is available in Additional file 3

Since 2000, WHO integrated guidelines for managing complications in pregnancy and childbirth, called IMPAC [14], have been the internationally prominent obstetric standards for low income settings, underlying multiple training and intervention programmes [19–21]. They were developed and peer-reviewed by expert panels without field testing [14]. Later on, focus has increased on the need for contextually-tailored interventions to achieve effective implementation [6, 22]. However, since the IMPAC guidelines’ first publication 16 years ago, a systematic literature search revealed that hardly any reviews on use and impact at the targeted low resource facilities have been conducted (Fig. 1). Simultaneously, quality assurance studies from low income settings call for more simple and achievable guidelines [23–28]. A review of Uganda’s 137 health sector guidelines found lack of involvement of end users in the development process to be a key contributor to ineffective, impractical, unclear, or too complex recommendations [18].

The resource constrained referral hospital of Zanzibar is an example of this struggle. There is ample room for improvement in intrapartum care, and fundamental shortages in number and knowledge level of birth attendants, space and equipment, guidelines use, and accountability measures [12]. Within this context, the PartoMa project aimed at developing locally agreed, achievable, and acceptable intrapartum guidelines, supporting partograph use and based on evidence, but carefully modified to local reality. We here describe the participatory development process and perceptions among staff 12 months post-implementation.

## Methods

The PartoMa study took place at the government-run Mnazi Mmoja Hospital in Zanzibar, Tanzania. This is the only referral facility for the archipelago’s population of 1.4 million, with 11–13,000 births and 50 maternal deaths annually. Our baseline study revealed a stillbirth rate of 59 per 1000 total births, of which half occurred intrapartum after admission to the hospital [12]. The average ratio of birth attendant to labouring women is 1:4 at daytime and 1:6 during evenings and nights. Notably, 30% of birth attendants are inexperienced intern doctors conducting their initial six-weeks obstetric rotation. When commencing the PartoMa project in 2014, obstetric guidelines were not routinely used, the locally promoted WHO composite partograph was hardly ever applied properly, and labour care was characterized by delays and inadequate management [12].

### Guidelines development process

The goal was to modify international guidelines to ensure achievability and unambiguousness in its use at the resource constraint facility. More specifically, patient load, staff numbers, supplies, and knowledge level of staff should be taken into account, and the guidelines should assist the providers in prioritizing surveillance and procedures in the best possible way, for individual labouring women as well as across the needs at the labour ward. Thereby, we hypothesized that the guidelines would be accepted and used by staff.

In November and December 2014, an initial version of the guidelines was drafted by four members of the study team (NM, JvR, TM, and BBN). TM is consultant obstetrician at the study site, and all four have obstetric experience in low income countries. WHO IMPAC guidelines [14] were applied as the frame for the development process, but supplemented and cross-checked by other evidence-based guidelines [29–35]. When modifications were made to well-established international recommendations, a systematic literature search was conducted in PUBMED for an overview of related scientific evidence.

Afterwards, an elaborate modification process was sketched, including testing and feedback cycles by both local birth attendants applying the guidelines in their clinical work and external specialists. The external peer review panel included four midwives and three obstetricians with elaborate clinical experience in low-income settings.

### Implementation process

Training in and awareness of the new guidelines were strengthened by associated PartoMa seminars, which were held quarterly, and have been continued by local staff after finalizing this study. The seminars provide case-based training at five work stations concerned with central topics in the guidelines. They are held in a communal room at the hospital and facilitated by hospital staff and members of the study team. Each seminar lasts four hours, commences after work, and is conducted twice. The strategy is to motivate individual providers to improve their quality of care voluntarily. No per diems are paid, and facilitators work voluntarily. Free lunch and guidelines booklets are provided.

In addition, the guidelines are available on posters in the maternity ward and often used during discussions of intrapartum care at the department’s daily clinical meetings.

### Staff’s perceptions and use

Twelve months after implementation, all birth attendants were requested to fill in an anonymous questionnaire on satisfaction with and use of the new guidelines, which is available in Additional file 1. Respondents included nurse-midwives and doctors in permanent positions at the Department of Obstetrics, as well as intern doctors. A five-point Likert scale was applied (1 = strongly disagree, 5 = strongly agree), and questions were in English and Swahili. Free text comments were welcomed on facilitators and barriers for use and recommendations for improvements. The questionnaire was modified from a previous Tanzanian study in which it was also found useful in evaluating a clinical educational intervention [36]. Two maternity theatre nurses not included among the study participants confirmed that the questionnaire was understandable. Questionnaire responses in Swahili were translated into English by RSK and all were entered electronically. Data was analysed by descriptive statistics.

## Results

### Development process

**Fig. 2:**
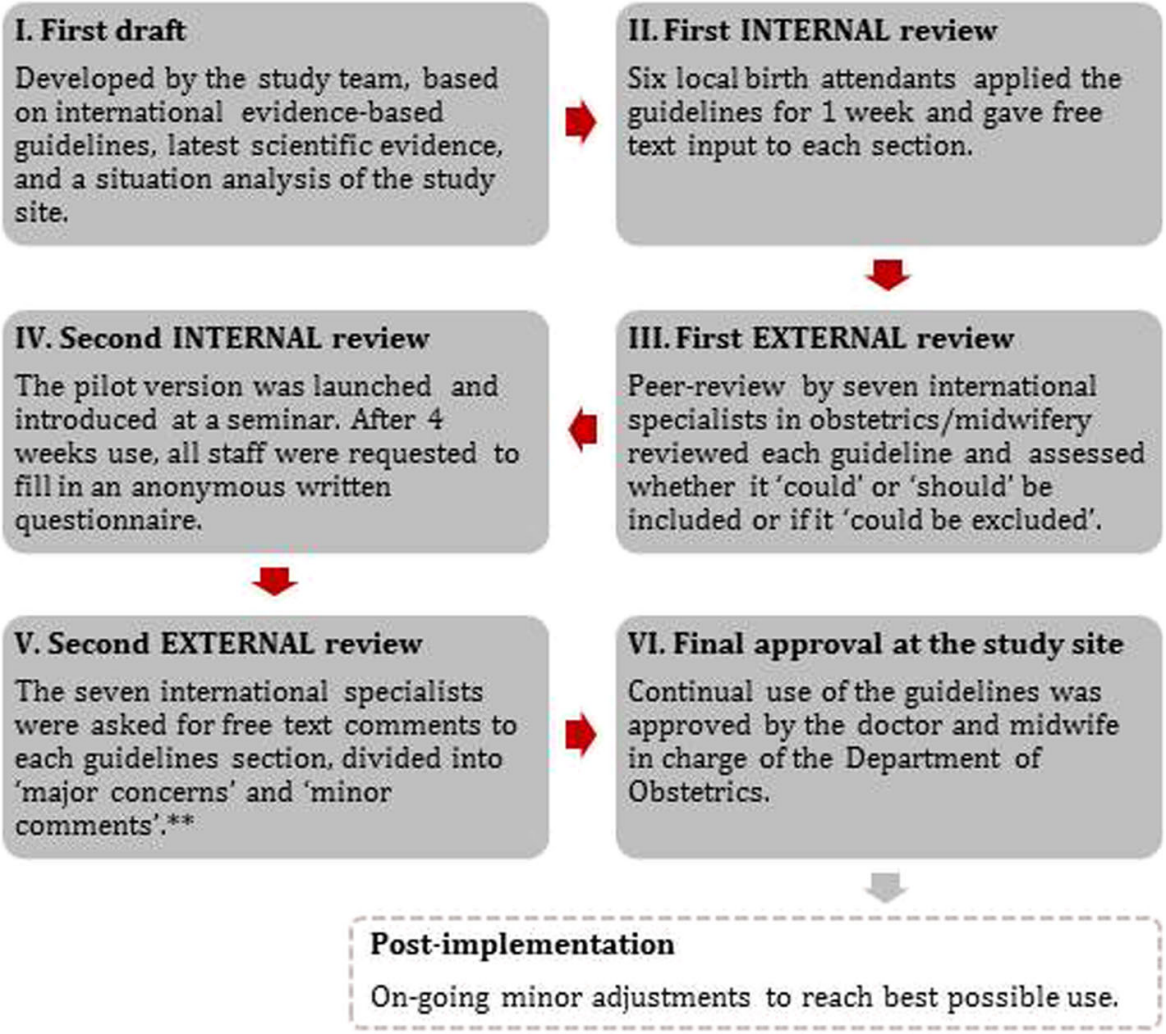
The six-steps participatory and internationally peer-reviewed development process of the PartoMa guidelines. ** Major concerns: If the reviewer feared that a specific guideline or graphic presentation could be dangerously used or misunderstood in clinical work. Minor comments: Any additional ideas for changes, including the graphical presentation, typos, etc.

Figure 2 provides an overview of what was eventually a six steps development process. By the end of December 2014, the initial guidelines draft (step I) was evaluated by six local staff, including the doctor and midwife in charge, another experienced nurse-midwife, two young medical doctors in permanent positions, and one intern doctor (step II). They applied the guidelines during 1 week’s work and handed in written free text evaluations of each page. Overall, they found the draft understandable and useful, but multiple minor comments relating to content, wording, and graphical presentation were taken into account.

A first external peer-review was then conducted by the seven international specialists (step III). They received the guidelines both integrated graphically and in spread sheets with references and the rationale for modifications made. They were asked to evaluate if each guideline ‘could’ or ‘should’ be included or if it ‘could possibly be left out’. Additional free text comments were welcomed. There was considerable diversity in comments, which resulted in discussions at length among the study team’s specialists.

By the end of January 2015, a modified pilot version was introduced at the first round of PartoMa seminars and a 4 weeks testing conducted (step IV). This was followed by a semi-structured questionnaire of staff, similar to the 12-months evaluation. On the two evaluation days, 32/46 (70%) of the participants from the launching seminars were present at work. Response rate among these was 100%. They found the pilot version useful, achievable, and understandable, and the evaluation only led to minor changes, primarily in wording.

The pilot-tested and re-modified version was thereafter sent for the second external peer-review (Step V). This time, the seven specialists were asked to comment in free text only, but divide their feedback into ‘major concerns’ (regarding a specific guideline or graphic presentation, which might be dangerously used in clinical work) and ‘minor comments’ (e.g. ideas for changes to graphics or wording). They raised no major concerns, and in March 2015 the guidelines were finally approved for internal use by the department’s doctor and midwife in charge (Step VI).

In addition, through on-going/recurring stays and work in the department by three members of the study team (NM, NH, and TM), participatory observations and informal discussions with staff were taken into account in order to adjust the guidelines to reflect reality as accurately as possible. This was applied both during the development process and during continuous post-implementation-optimization of the guidelines. It mainly included simplifications of language and graphical presentations.

### Guideline content

**Fig. 3:**
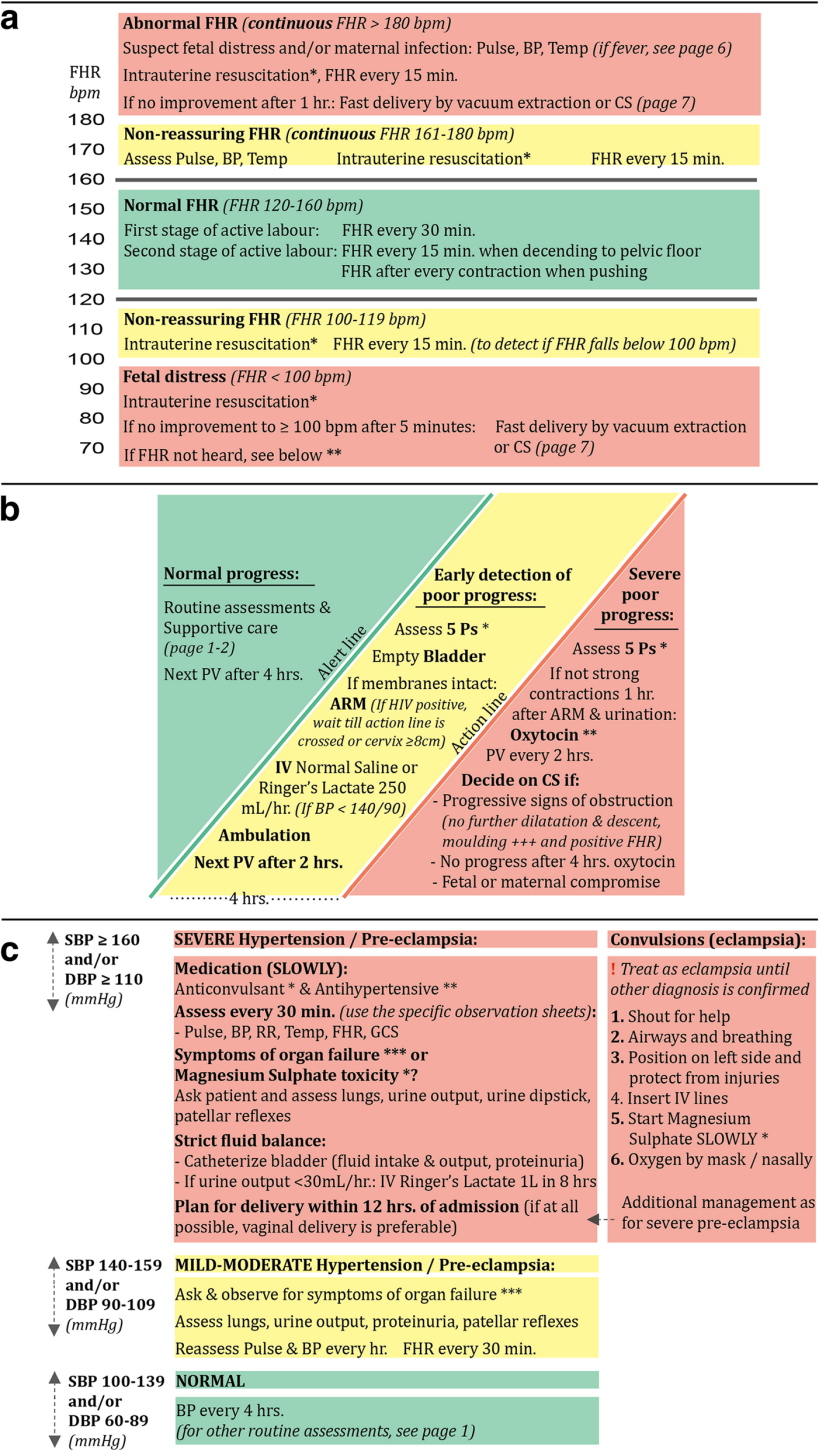
The three parts of the PartoMa guidelines most appreciated by staff: (**a**) Management of abnormal foetal heart rate; (**b**) Management of poor progress in first stage of active labour (cervical dilatation ≥4 cm and regular painful contractions); (**c**) Management of hypertensive disorders. The graphics are based on the WHO partograph, and the star symbols (*) refer to recommendations further described on the page below in the guidelines. A full overview of the PartoMa guidelines is available in Additional file 2. ARM, artificial rupture of membranes; BP, blood pressure; bpm, beats per minute; CS, caesarean section; FHR, foetal heart rate; PV, vaginal examination; Temp, temperature. *© 2015 The PartoMa Study, University of Copenhagen. All Rights Reserved*

The development process resulted in the eight-paged PartoMa guidelines booklet on partograph-associated decision-support for common intrapartum management. The booklet is available in Additional file 2. It contains the following sections: routine assessments and supportive care during labour, delivery, and the first 2 h postpartum/neonatally; management of the most common intrapartum complications related to foetal heart rate (FHR), labour progression, and maternal condition (hypertensive disorders, hypotension, and fever); decision-to-delivery intervals for caesarean section; and vacuum extraction. To strengthen linkage to the WHO partograph, graphical presentations were developed based on the partograph’s graphics (Fig. 3). As major deficiencies existed in basic labour care, both basic and emergency management were included [12]. Colour codes were applied as indicators of urgency (green, yellow, red). It was emphasized that while guidelines represent the best possible management for the majority of cases, there may be situations where alternative practice is preferable, and in such cases, management should always be discussed with colleagues. The final booklet fits into staff uniform pockets (10 × 15 centimetres each).

Three pages in the guideline were the most appreciated among staff (Table 1), and the main parts of these are presented in Fig. 3. Presentation of perspectives raised by staff, external reviewers, and study team members is delimited to these three pages.

**Table 1:** 12 months evaluation of use and satisfaction with the PartoMa guidelines: Background characteristics of respondents, own use, and favorite guideline page(s)

	Doctors^a^	Nurse-midwives^a^	Intern doctors^b^
	*n = 12*	*n = 23*	*n = 49*
N (%)			
Years of obstetric/midwifery experience			
< 1 year	5 (41.7%)	5 (21.7%)	49 (100.0%)
1–5 years	6 (50.0%)	14 (60.9%)	0 (0.0%)
> 5 years	1 (8.3%)	4 (17.4%)	0 (0.0%)
Use of the PartoMa guidelines			
Every day when at work	11 (91.7%)	17 (73.9%)	43 (87.8%)
At least once a week	1 (8.3%)	3 (13.0%)	5 (10.2%)
Less than once a week	0 (0.0%)	1 (4.3%)	1 (2.0%)
Never	0 (0.0%)	2 (8.7%)	0 (0.0%)
PartoMa seminars attended			
0	1 (8.3%)	3 (13.0%)	9 (18.4%)
1	3 (25.0%)	12 (52.2%)	17 (34.7%)
≥ 2	8 (66.7%)	7 (30.4%)	23 (47.0%)
Information missing	0 (0.0%)	1 (4.3%)	0 (0.0%)
Favourite part(s) of the PartoMa guidelines			
Routine surveillance & supportive care	7 (58.3%)	16 (69.6%)	32 (65.3%)
Fetal heart rate and fetal distress	10 (83.3%)	17 (73.9%)	32 (65.3%)
Labour progression and poor progress	5 (41.7%)	17 (73.9%)	26 (53.1%)
Hypertensive disorders	9 (75.0%)	14 (60.9%)	39 (80.0%)
Fever, high pulse, low blood pressure	5 (41.7%)	7 (30.4%)	21 (43.0%)
Vacuum extraction^c^	8 (66.7%)	5 (21.7%)	24 (49.0%)

^a^All doctors and nurses/midwives in permanent positions at the obstetric division of the department by the end of January and beginning of February 2016 were requested to fill in the questionnaire (response rates: 92.3% and 88.5%, respectively). Doctors included 11 medical doctors and 1 assistant medical doctor

^b^All intern doctors who had conducted their six weeks obstetric clinical rotation since March 2015 were requested to fill in the questionnaire (response rate: 80.3%). At the time of data collection, some had finalized their internship and left for positions outside Zanzibar, and they could therefore not be reached

^c^This section was reproduced from the Advanced Life-saving Skills in Obstetrics’ course syllabus, with permission from their legal board [33]

### A. Abnormal foetal heart rate (FHR)

No studies exist that compare different FHR auscultation intervals [37]. During first stage of active labour, the international benchmark of 30 min intervals was often hard to achieve, and 1 h was included as a minimum acceptable interval. For second stage, due to high numbers of intrapartum stillbirths, and known risks of pushing on the foetus’ oxygen supply, the importance of close monitoring was stressed (Additional file 2, page 1) [12, 32].

While it has internationally been suggested that FHR of 110–160 beats per minute (bpm) is normal, evidence is scarce [38]. Therefore, non-reassuring zones for both low and high FHR were kept in alignment with IMPAC and the partograph currently applied (100–119 bpm and 161–180 bpm; Fig. 3a) [14]. There was previously a common understanding among staff that FHR <120 or >160 bpm was an indication for caesarean section. Simultaneously, even for FHR <100 bpm, it was common to wait 30 min before deciding for caesarean section, which was often followed by delays in the decision-to-delivery interval [12]. Likewise, IMPAC is unclear on this matter [14]. Management in the non-reassuring zones was now specified and did not include operative delivery (Fig. 3a). For FHR < 100 bpm, we agreed on an interval of 5 min before re-check and, if FHR remaining <100 bpm, plan for expedite delivery (in the second stage of labour preferably by vacuum extraction). Concerning FHR >180 bpm, no evidence or international consensus was found for when to decide on caesarean section/vacuum extraction. Consensus was reached on a 1 h time frame (Fig. 3a).

### B. Poor progress in first stage of active labour

For routine surveillance during active labour, IMPAC’s recommendations for vaginal examination were found achievable (Additional file 2, page 1) [14]. Assessments of contractions half hourly was, however, structurally impossible; one birth attendant would have a full time job assessing contractions on three labouring women. We found no studies comparing different frequencies of monitoring contractions. When progress is normal, no oxytocin administered, and maternal and foetal conditions reassuring, consensus was reached on counting contraction every second hour. Evaluating foetal head descent was reduced from every two to every 4 h.

Management of poor progress in first stage of active labour is illustrated by WHO’s alert and action lines (Fig. 3b). A similar diagram was successfully used in a previous Tanzanian study [25]. Before guidelines implementation, more than 20% of labouring women received oxytocin for augmentation, often on doubtful indication, and oxytocin was a predisposing factor for stillbirth [12]. To ensure safe administration of this potent drug in the resource constrained context, a restrictive regimen was agreed upon where oxytocin is saved for women crossing the action line with ruptured membranes for ≥1 h and <4 strong contractions in 10 min [39]. Danger of uterine hyperstimulation was stressed, and a restrictive dose recommended: 2.5 units in 500 ml Ringer’s Lactate/Normal Saline at 10 drops per minute, infusion increased with 5 drops per minute every 30 min until 4–5 strong contractions per 10 min. By a ‘5 Ps mnemonic’, elaborated from the Advanced Life-saving Skills in Obstetrics (ALSO) course [33], attention was drawn to alternative and less dangerous interventions to augment labour: i.e. artificial rupture of membranes, emptying bladder, exercise, oral intake, continuous support, and intravenous normal saline or Ringer’s Lactate (Additional file 2, page 4) [14, 40].

When the action line is crossed, we found no clear evidence for when to decide in favour of caesarean section. However, women often suffered from severe delays in management of poor progress in this facility [12]. Consensus was reached on three indications (Fig. 3b).

### C. Hypertensive disorders

This part provides management guidance for hypertension and pre-eclampsia/eclampsia (Fig. 3c; Additional file 2, page 5). In accordance with the scope of the guidelines, recommendations on pre-labour management were not included. Diagnostic criteria for pre-eclampsia were based on RCOG guidelines, but for simplicity mild and moderate pre-eclampsia were merged, and biochemical/haematological impairment was excluded from the definition of severe pre-eclampsia (Additional file 2, page 5) [31]. Due to time and budget constraints, urine dipstick was excluded from routine assessments of labouring women and saved for women with hypertensive disorders or signs of urinary tract infection.

Management of hypertensive disorders was inspired by the LIVKAN treatment chart, which was found useful among birth attendants in Somali-land [34]. For simplicity, management of severe hypertension and severe pre-eclampsia/eclampsia were grouped together. Furthermore, for these severe cases an intensive treatment protocol was agreed upon aiming at delivery within 12 h of admission, versus 24 h in international guidelines [14, 31, 41]. This was because many women were admitted with severe hypertension of unknown duration (Fig. 3c) [12]. Medical treatment was restricted to the drugs available at the study site (magnesium sulphate as anticonvulsant and the antidote calcium gluconate; hydralazine for fast antihypertensive treatment). Concerning hydralazine, its associations with maternal hypotension, placental abruption, and adverse perinatal outcome were considered when deciding on a more conservative regimen than suggested by IMPAC [14, 42]: 5 mg every 20 min until systolic blood pressure < 160 mmHg (Additional file 2, page 5) [35].

### Staff’s perceptions and use

The questionnaire evaluation of the guidelines was responded to by 12/13 (92%) doctors and 23/26 (89%) nurse-midwives in permanent positions at the department, as well as by 49/61 (80%) intern doctors (Table 1).

**Fig. 4:**
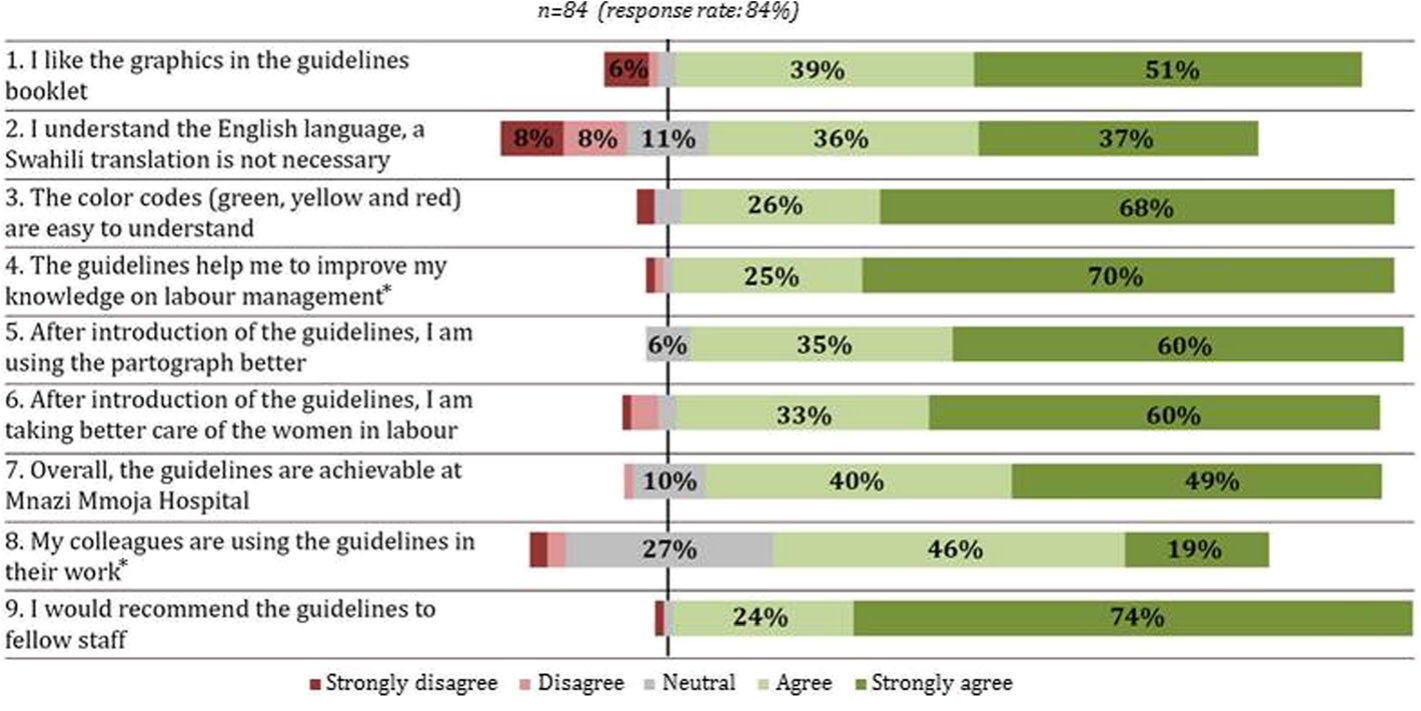
Five-point Likert scale evaluation of health providers’ perceptions and use of the PartoMa guidelines 12 months after implementation. The respondents included 12 medical doctors and 23 nurse midwives in permanent positions at the Department of Obstetrics, as well as 49 intern doctors who had conducted their six-weeks obstetric rotation during the past ten months. Nurse-midviwes disagreed more often to question 2 (mean score 3.30), when compared to intern doctors (mean score 3.94) and doctors in permanent positions (mean score 4.50). Otherwise, no major differences were found between the groups. * Concerning questions 4 and 8, 1 (1%) and 2 (2%) health providers, respectively, did not respond

The Likert scale evaluation is presented in Fig. 4. Among all staff, there was agreement that the PartoMa guidelines were achievable at the study site (mean score 4.22–4.65), and the graphics and colour codes for urgency appeared broadly accepted (mean scores 4.22–4.50 and 4.39–4.75, respectively). The majority stated that the guidelines helped to improve knowledge on labour management (mean score 4.32–4.78), and that they were providing better care to labouring women by using the guidelines (mean score 4.22–4.58). Staff would recommend the guidelines to colleagues (mean score 4.48–4.92). Particularly some nurse-midwives indicated that the English language was a challenge in understanding the guidelines (mean score 3.30–4.50).

All cadres of staff found ‘FHR and foetal distress’ (Fig. 3a) to be one of the most useful pages. For nurse-midwives, the ‘labour progress and poor progress’ page was also a favourite (Fig. 3c), while doctors and intern doctors were particularly fond of ‘hypertensive disorders’ (Fig. 3b; Table 1).

Doctors, including interns, more often responded to use the guidelines on daily basis than nurse-midwives (54/61 (89%) and 17/23 (74%); Table 1). Similarly, when asked about colleagues’ use, the mean Likert scale score indicated that some staff did not apply the guidelines on regular basis (3.67–4.05, Fig. 4).

In free text comments, some respondents described how there were no barriers for them to use the guidelines; in particular intern doctors emphasized how the PartoMa guidelines were their “friend in the pocket”. Others referred to the high work load being a challenge for guidelines use. Many suggested that the combination of guidelines and seminars should continue beyond the study period, and that the intervention package would also be useful at other Zanzibarian facilities. Often, additional topics were suggested for inclusion in the guidelines; e.g. induction of labour and management of postpartum haemorrhage.

## Discussion

We here presented a participatory approach to development of innovative, readily available, integrated guidelines for intrapartum management, suited for birth attendants at the referral hospital of Zanzibar. As often reported from similar settings, intrapartum guidelines were not routinely used prior to the study, and quality of surveillance and decision-making was poor [12, 18, 23–25, 43, 44]. The development process revealed that international recommendations were often too time- and resource-consuming, as well as underspecified, too complex, and too long, leading to demoralized attitudes. Twelve months post-implementation, staff found that the PartoMa guidelines ensured achievable, acceptable, and applicable decision-support for timely surveillance, treatment, and triage; they felt that the guidelines help them to provide better care.

### Interpretation

The guidelines development process was highly dependant on time, project funding, access to evidence, capacity to synthesize and apply evidence, skills in graphical design, and a robust coordination of partners; resources that can seldomly be spared at facilities like the study site. Likewise, review of Uganda’s health sector guidelines concluded that low income countries face multiple barriers in conducting guidelines modification processes [18]. It is therefore warranted that the central development process of international evidence-based guidelines targeting low resource settings involve end users already in the initial phases.

During the initial steps of development, the PartoMa guidelines appeared to better match the reality that birth attendants experience. This may be associated with early involvement of end users and the study team’s familiarity with the context. Furthermore, the initial guidelines draft provided a new transparency in what was expected best practice at the study site. This enabled formulation of audit standards for the simultaneous baseline study on quality of care [12]. During the later steps of guidelines adjustments, feedback from criteria-based audit aided in further prioritising key contents of the guidelines. As commented elsewhere, production of contextually tailored guidelines for low income settings will be the first step for conducting comprehensive audits [45].

Non-realistic guidance may lead to either no use or unpredictable individual adaptations, and both scenarios may cause variable and riskful performance [46]. More specifically, studies on clinical guidelines show that standards that are simple and easy to understand have a greater chance of implementation [47]. In the PartoMa study, the guidelines’ restriction to eight pages, simplified wording, partograph-associated graphical presentations, and colour codes for urgency appeared to be key drivers for acceptance and use. It was challenging to dare modifying well-established evidence-based guidelines to reach simplicity and achievability. However, the diversity in the peer-reviewers’ comprehensive comments, and the often limited scientific evidence base, eased the process [30].

Even when simplified and only taking up eight pages, PartoMa guidelines cover an integrated continuum of common care for women giving birth. First, recommended monitoring and treatment of the individual woman took into account that each birth attendant on average cared for 4–6 women simultaneously. Second, because severe obstetric complications are often preceeded by delays in basic care, we found it crucial to integrate routine and emergency obstetric care [12]. Third, we emphasized partograph use as an integrated early warning tool to assess FHR, labour progress, and maternal condition throughout latent and active phase of labour, including the often forgotten second stage [32].

Guidelines development is an on-going process. While unnecessary updates and changes cause pointless confusion, it is crucial ethically to ensure updates in relation to emerging scientific evidence and changes in supplies, staff numbers, and knowledge level of staff. In addition, during the first implementation year, in alignment with suggestions from staff and observations of care, we have continuously incorporated minor adjustments in wording and graphical presentations to improve unambiguousness and clinical relevance. A similar strategy has proven highly cost-effective in a high-income setting, where continual modifications of guidelines were conducted in response to practice variances [48]. Moreover, we plan for a future second edition, which include additional management recommendations as suggested by staff; e.g. induction of labour, trial of scar, management of postpartum bleeding, and neonatal resuscitation. Lastly, we hope to introduce a Swahili version.

### Strengths and limitations

This pragmatic development process was designed to suit an obstetric department with severe resource and capacity constraints [12]. Through the in-depth peer-review process, we believe to have taken all reasonable precautions to verify the information contained in the PartoMa guidelines. Importantly, the guidelines were developed specifically to guide staff at the study site. If implemented in better resource settings, the recommendations might cause unintended effects, e.g. unnecessarily infrequent assessments. Notably, the group of birth attendants at the study site was dominated by rather inexperienced professionals. This was due to the immense responsibility given to intern doctors, and to a high turnover among doctors and nurse-midwives in permanent positions, which are typical challenges in East African settings.

The intervention design does not allow clear differentiation of staff’s perceptions of the guidelines versus the seminars. However, 54% of the respondents in the 12-months evaluation had attended ≤1 seminar, and it seems unlikely that their perceptions were primarily based on the seminars. Moreover, in line with the call for achievable guidelines from similar settings, the major modifications here described seem crucial for acceptance and use [18, 23–25].

Restricting pilot-testing and 12-months evaluation to written questionnaires may have been too narrow to capture all dimensions.

## Conclusions

This participatory, peer-reviewed guideline development process is a crucial example of bridging the gap between evidence-based international recommendations and local realities at resource limited health facilities. The PartoMa guidelines ensured a readily available, achievable, and acceptable decision-support for timely surveillance, treatment, and triage during labour. It is, however, unlikely that all resource limited facilities will have the means for such major development processes. It is warranted that future international guidelines targeted at low income countries take into account the realities of care-giving in such contexts; if easy usability is not ensured, simply producing evidence-based guidelines will not drive change. Preliminary findings regarding effects of the PartoMa guidelines on clinical practice and labour outcome are promising and will be presented in another paper. It will furthermore be relevant to investigate the PartoMa guidelines’ feasibility in similar settings.

## Additional files

Additional file 1: The 12-months evaluation questionnaire of satisfaction with and use of PartoMa guidelines. (PDF 351 kb)

Additional file 2: The PartoMa guidelines booklet. (PDF 1608 kb)

Additional file 3: A systematic literature search: Labour and delivery guidelines for African low-income settings. (PDF 401 kb)

## Abbreviations

ARM: artificial rupture of membranes
BP: blood pressure
Bpm: beats per minute
CS: caesarean section
FHR: foetal heart rate
G: grams
IMPAC: WHO integrated guidelines for managing complications in pregnancy and childbirth
PV: vaginal examination
Temp: temperature
WHO: World Health Organization
