# Supplementary material for: Labour management guidelines for a Tanzanian referral hospital: The participatory development process and birth attendants’ perceptions
Source: BMC Pregnancy Childbirth. 2017 Jun 7;17:175. doi: 10.1186/s12884-017-1360-2 (PMC5463375; doi:10.1186/s12884-017-1360-2)
Supplement: Supplementary file 1 — The 12-months evaluation questionnaire of satisfaction with and use of PartoMa guidelines. (PDF 351 kb) [file 12884_2017_1360_MOESM1_ESM.pdf]

The PartoMa Guidelines Booklet Evaluation *(Tathmini ya kitabu PartoMa Guidelines kwa watumiaji)*

This questionnaire is anonymous and will help us make the PartoMa guidelines as useful as possible.
(Dodoso hii ni bila majina ni kwaajili ya kutusaidia kufanya miongozo PartoMa kuwa muhimu kadri iwezekanavyo.)

Employment *(Aina ya muajiriwa)*: ◯ Nurse/midwife ◯ Intern doctor ◯ AMO ◯ Registrar doctor ◯ Other: ____________

In the last 6 months, which areas have you worked with (you may 'X' several fields)?
(Miezi 6 iliopita, maeneo gani umefanya kazi? Unaweza kuweka 'X' eneo zaidi ya moja)

◯ Antenatal care ◯ Labour and delivery care
◯ Post-delivery care ◯ Theater, caesarean sections
◯ Gynaecological ward

Did you attend any of the PartoMa seminars (you may 'X' several fields)?
(Umewahi kuhudhuria semina yoyote ya PartoMa? Unaweza kuweka 'X' eneo zaidi ya moja)

◯ 1st seminar (January 2015) ◯ 2nd seminar (March 2015)
◯ 3rd seminar (June 2015) ◯ 4th seminar (October 2015)
◯ 5th seminar (January 2016) ◯ No, I did not attend any of the PartoMa *(Hapana, sijahudhuria semina yoyote ya PartoMa)*

How many years of experience do you have with labour care? *(Miaka ya uzoefu wa kazi ya ukunga)*

◯ less than 1 year *(chini ya mwaka moja)* ◯ 1-5 years *(Mwaka 1-5)* ◯ 6-10 years *(Miaka 6-10)* ◯ more than 10 years *(zaidi ya miaka 10)*

Circle one correct number for each item *(Zungushia jibu moja sahihi kwa kila swali)*:
1) Strongly disagree *(Nakataa kabisa)*
2) Disagree *(Nakataa)*
3) Neither agree or disagree *(Sikubali wala sikatai)*
4) Agree *(Nakubali)*
5) Strongly Agree *(Nakubalikabisa)*

	**Disagree** *Kataa* ◄———► **Agree** *Kubali*
**Overall impression of the PartoMa guidelines** *(Hisia za jumla kwa kitabu)*	
1. I like the graphics in the guidelines booklet. *(Nimependa mpangilio katika miongozo ya kijitabu)*	1 2 3 4 5
2. I understand the English language in the guidelines – a Swahili translation is <u>not</u> necessary. *(Nimeielewa lugha ya Kiingereza katika miongozo – kutafsiriwa Kiswahili siyo lazima)*	1 2 3 4 5
3. The color codes (green, yellow and red) are easy to understand. *(Ishara za rangi (kijani, njano na nyekundu) ni rahisi kuelewa)*	1 2 3 4 5
4. The guidelines help me to improve my knowledge on labour management. *(miongozo inanisaidia kuboresha ufahamu wangu juu ya utekelezaji wa kazi ya uzalishaji)*	1 2 3 4 5
5. After introduction of the PartoMa guidelines, I am using the partogram better. *(Baada ya kuanzishwa kwa miongozo ya ParToma natumia patogram vizuri zaid)*	1 2 3 4 5
6. After introduction of the PartoMa guidelines, I am taking better care of the women in labour. *(Kazi yangu ya uzalishaji imeboreka kwa kutumia miongozo ya kijitabu cha miongozo)*	1 2 3 4 5
7. Overall, the guidelines are achievable at Mnazi Mmoja Hospital. *(Kwa ujumla, miongozo ni mafanikio katika Hospitali ya Mnazi Mmoja)*	1 2 3 4 5
8. My colleagues are using the PartoMa guidelines in their work. *(Wafanyakazi wenzangu wanatumia miongozo ya PartoMa katika kazi zao)*	1 2 3 4 5
9. I would recommend the PartoMa Guidelines to fellow staff *(Nitapendekeza PartoMa guidelines kwa wakunga wengine)*	1 2 3 4 5

Use of the PartoMa guidelines *(Matumizi yako ya kijitabu cha muongozo)*

10. I have used the guidelines in my work at the maternity ward?
(Nimekua nikitumia kijitabu chamiongozo katika kazi yangu)

◯ Yes *(ndiyo)* ◯ No *(hapana)*

11. I use the guidelines this often:
(Natumia miongozo kama ifuatavyo)

◯ Every day when I am on work in the maternity ward *(Siku zote niwapo kazini)*
◯ At least once a week *(kwa uchache mara 1 katika wiki)*
◯ Less than once a week *(chini ya mara1 lwa wiki)*
◯ Never *(sijawahi kutumia)*

12. What part or parts of the guidelines do you use most often (you may 'X' several fields)?
(Ni sehemu ipi au zipi katika miongozo unaitumia zaidi, unaweza kuweka 'X' eneo zaidi ya moja)

◯ Introduction (page 0, *ukurasa 0*)
◯ Routine assessments and supportive care (page 1-2, *ukurasa 1-2*)
◯ Fetal heart rate and liquor (page 3, *ukurasa 3*)
◯ Vaginal examination and contractions (page 4, *ukurasa 4*)
◯ High blood pressure and proteinuria (page 5, *ukurasa 5*)
◯ Low blood pressure, high pulse & high temperature (page 6, *ukurasa 6*)
◯ Caesarean section and vacuum extraction (page 7, *ukurasa 7*)

13. Why may it be difficult for you and your collegues to use the partogram in your work? Please describe in free text, in English or Swahili:

(Kwanini inakuwa ngumo kwako kutumia partogram katika sehemo ya kazi? Tafadhali kuwa huru andika kwa kiingereza au Kiswahili)

14. Why may it be difficult for you and your colleagues to use the PartoMa Guidelines in your work? Please describe in free text, in English or Swahili:

(Kwanini inakuwa ngumo kwako kutumia PartoMa Guidelines katika sehemo ya kazi? Tafadhali kuwa huru andika kwa kiingereza au Kiswahili)

Final question *(Swali la mwisho)*

15. Do you have any general suggestions on how the PartoMa guidelines can be improved, or other comments? Please write any comments below in English or Swahili.

(Je, una mapendekezo yoyote kwa ujumla juu ya jinsi miongozo PartoMa inaweza kuboreshwa? Tafadhali andika maoni yoyote chini kwa Kiingereza au Kiswahili)

Thank You very much! Asante sana!
