# Supplementary material for: Labour management guidelines for a Tanzanian referral hospital: The participatory development process and birth attendants’ perceptions
Source: BMC Pregnancy Childbirth. 2017 Jun 7;17:175. doi: 10.1186/s12884-017-1360-2 (PMC5463375; doi:10.1186/s12884-017-1360-2)
Supplement: Supplementary file 2 — The PartoMa guidelines booklet. (PDF 1608 kb) [file 12884_2017_1360_MOESM2_ESM.pdf]

PartoMa Guidelines

Partogram-Associated
Labour Management Guidelines

Version 1.2 | Launched in March 2015
Latest minor revisions in wording/graphics: January 2016

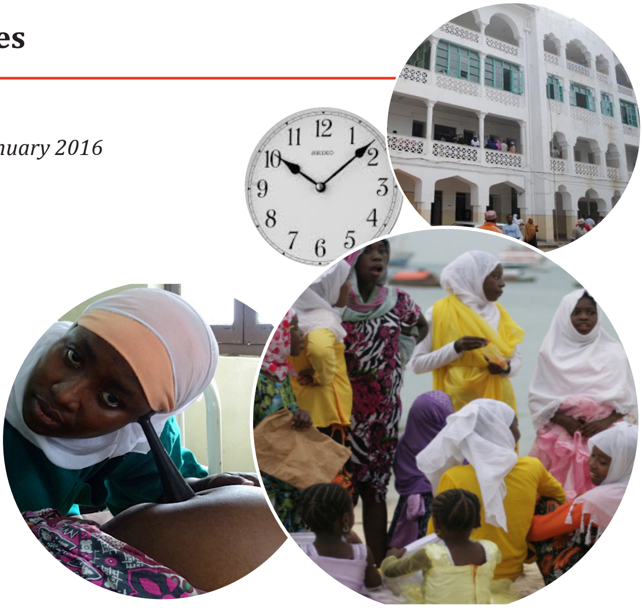

The PartoMa Project
Department of Obstetrics &
Gynaecology,
Mnazi Mmoja Hospital

© 2015 The PartoMa Study, University of Copenhagen. All Rights Reserved.

Version 1.2

INDEX - *and example of a correctly used partogram*

I. ROUTINE ASSESSMENTS & SUPPORTIVE CARE: Page 1 - 2

II. PARTOGRAM-ASSOCIATED MANAGEMENT: Page 3 - 6

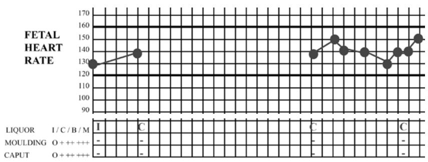

Fetal heart rate & Liquor: ***Page 3***

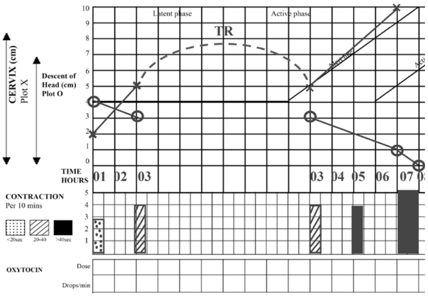

Vaginal examination & contractions ***Page 4***

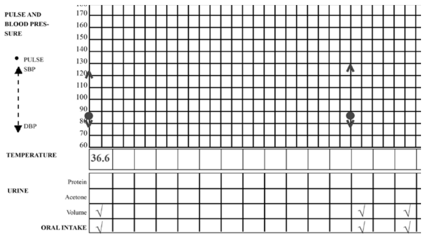

High blood pressure & Proteinuria: ***Page 5***

Low blood pressure, High pulse & High temperature ***Page 6***

III. CAESAREAN SECTION & VACUUM EXTRACTION: Page 7

COLOUR CODES

NORMAL: Routine assessments & supportive care

WARNING: Attention & Treatment

DANGER: **IMMEDIATE ACTION!**

REMEMBER

The partogram must be used in the care for ALL women in labour *(unless immediate need for intervention on admission)*

The partogram is a decision support tool: Each assessment requires analysis of the partogram as a whole by

3 DIAGNOSTIC QUESTIONS:

1. Is **mother** in a good condition?
2. Is **baby** in a good condition?
3. Is **progress** normal?

If any of these is abnormal, consult with guidelines and/or a senior colleague

! *The PartoMa guidelines represent the best possible management for the majority of patients, but there may be situations where alternative management is preferable. In such cases, treatment should be discussed with colleagues.*

ABBREVIATIONS

ARM, artificial rupture of membranes
BP, blood pressure
Bpm, beats per minute
CPD, cephalopelvic disproportion
CS, caesarean section
DBP, diastolic blood pressure
E.g., for example
FHR, fetal heart rate
GCS, Glasgow Coma Scale
Hr., hour
Im, intramuscular
Iv, intravenous
Min., minutes
MmHg, millimetres of mercury
PROM, premature rupture of membranes
RR, respiratory rate
SBP, systolic blood pressure
SRM, spontaneous rupture of membranes
Temp, temperature
TR, transfer
UTI, urinary tract infection
PV, per vaginal examination
≤, less than or equal to
≥, greater than or equal to

REFERENCES

! *Adjustments have been made to reach best possible use at Mnazi Mmoja Hospital*

World Health Organization's "Managing Complications in Pregnancy and Childbirth: A Guide for Midwives and Doctors" (2007) was used as the main fraim, but supplemented by evidence-based guidelines from Royal College of Obstetrics and Gynaecology, International Federation of Gynecology and Obstetrics, the Advanced Life-saving Skills in Obstetrics course, the LIVKAN chart for pre-eclampsia/eclampsia, and the Safe Delivery App.

Version 1.2

© 2015 The PartoMa Study, University of Copenhagen

Version 1.2

UNCOMPLICATED LABOUR: ROUTINE ASSESSMENTS IN LATENT & ACTIVE PHASE

When maternal vital signs, FHR and progress are normal

ON ADMISSION

Obstetric history
Previous and present pregnancy

Initial assessments
FHR — *Lie/presentation/descent*
Pulse, BP — *Contractions*
Temp — *PV*

Obstetric risks needing extra attention?
E.g. maternal illness, previous CS, concerns for the baby, PROM, meconium stained liquor, vaginal bleeding, induction of labour

LATENT PHASE

Regular painful contractions & cervix < 4 cm

Every 4 hrs.
& when changes occur:
(E.g. rupture of membranes or increasing contractions)

Pulse, BP
FHR *
Abdominal exam *(lie/presentation?)*
PV **
Contractions ***

FIRST STAGE, ACTIVE PHASE

Cervix 4 - 9 cm ****

Every ½ hr. *(every 1 hr. as a minimum)*:
FHR*

Every 2 hrs.:
Contractions ***
Urine output *(encourage bladder emptying spontaneously)*

Every 4 hrs.:
PV **
Pulse, BP

SECOND STAGE, ACTIVE PHASE

Cervix fully dilated

Monitor FHR closely *:
Before pushing: Every 15 min.
When pushing: After every contraction

! *Assure emptying bladder before starting to push and every ½ hr.*

Contractions & PV **:
Every ½ hr.

Pulse & BP: As in first stage of labour

* AUSCULTATION OF FHR

Auscultate after a contraction for minimum 1 min.
Always assure that it is FHR and not maternal Pulse

** PV - What to assess?

Cervical dilatation and state of cervix *(effacement, thin/thick, rigid/soft, oedematous)*
Vagina *(warm+moist/hot+dry)*
State of membranes
Head descent *(in relation to ischial spines or fifths of head palpable)*
Colour of liquor if ruptured membranes
Presentation and position
Moulding & caput *(if membranes are ruptured)*

! ***No PV if placenta praevia is suspected*** *(page 3)*

*** CONTRACTIONS

Contractions are assessed by palpating the abdomen for 10 min. and carefully registering frequency and duration of each uterine tightening.

! ***Strong contractions are 3-5 contractions per 10 min., each lasting more than 40 sec.***

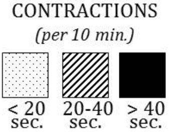

**** TRANSFER TO ACTIVE PHASE

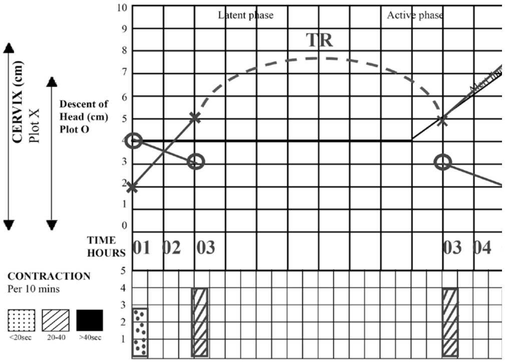

! ***Active phase starts when cervix is 4 cm dilated***

! ***If the patient is admitted in latent phase, when active phase starts, all observations must be transferred to the alert line***
(see also the partogram on page 0)

Version 1.2

 © 2015 The PartoMa Study, University of Copenhagen

Version 1.2

UNCOMPLICATED BIRTH: ROUTINE POST-DELIVERY CARE

AFTER DELIVERY OF BABY

Mother: **Active management of third stage** *(IM Oxytocin 10 units, controlled cord traction and uterine massage)*

Baby: **Apgar score *** *(1 and 5 min.)*
Skin-to-skin contact
Breastfeeding *(within 30 min.)*

AFTER DELIVERY OF PLACENTA

Mother: **Perineal/genital trauma?**
Placenta complete?

EVERY 15 MIN. IN THE FIRST 2 HRS.

Every 30 min. as a minimum

Mother: **General condition**
Uterine consistency *(height of fundus)*
Vaginal blood loss

Baby: **Breathing, colour & warmth**
Cord bleeding *(teach mother to help assessing)*

BEFORE DISCHARGE

Mother: **Pulse, BP, urination, vaginal discharge**

Baby: **Birth weight**

Give instructions:

1. Danger signs for mother & baby (fever, bleeding)
2. Information on any complications
3. Family planning

* APGAR SCORE

	0	1	2
Appearance	Blue/pale all over	Blue/pale limbs & pink body	Pink body & limbs
Pulse	Absent	< 100	≥ 100
Grimace	No response to stimulation	Grimace when stimulated	Cry when stimulated
Activity	None	Some flexion	All limbs flexed
Respiration	Absent	Weak	Strong

SUPPORTIVE CARE DURING LABOUR & DELIVERY

Routine care for ALL women in labour

1. Respect, empathy & caring support

2. Timely assessments, documentation & analyses of the partogram *(remember the 3 diagnostic questions, page 0)*

3. Clear, timely and supportive communication

4. Privacy and confidentiality

5. Cleanliness:
- Strict hand washing before and after procedures
- Gloves for all procedures, e.g. PVs
- Wash hands before and after PV
- Ensure cleanliness of birthing area & clean up spills immediately

! *Alcohol handrub is the best disinfectant*
! *Do not share towels or soap*

6. Ambulation (position of woman's choice)

7. Urination *(encourage spontaneous bladder emptying every 2 hrs.)*

8. Eating and drinking freely

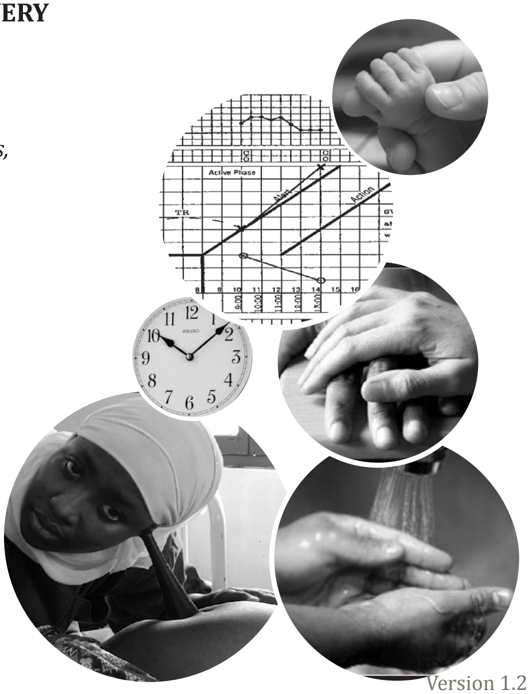

Version 1.2

© 2015 The PartoMa Study, University of Copenhagen

Version 1.2

FETAL HEART RATE (FHR)

FHR *bpm*

180 – **Abnormal FHR** ***(continuous** FHR > 180 bpm)*

Suspect fetal distress and/or maternal infection: Pulse, BP, Temp *(if fever, see page 6)*

Intrauterine resuscitation*, FHR every 15 min.

If no improvement after 1 hr.: Fast delivery by vacuum extraction or CS *(page 7)*

170 – **Non-reassuring FHR** ***(continuous** FHR 161-180 bpm)*

Assess Pulse, BP, Temp — Intrauterine resuscitation* — FHR every 15 min.

160

150 – 130 – **Normal FHR** *(FHR 120-160 bpm)*

First stage of active labour: FHR every 30 min.

Second stage of active labour: FHR every 15 min. when decending to pelvic floor
FHR after every contraction when pushing

120

110 – **Non-reassuring FHR** *(FHR 100-119 bpm)*

Intrauterine resuscitation* FHR every 15 min. *(to detect if FHR falls below 100 bpm)*

100

90 – 70 – **Fetal distress** *(FHR < 100 bpm)*

Intrauterine resuscitation*

If no improvement to ≥ 100 bpm after 5 minutes: Fast delivery by vacuum extraction or CS *(page 7)*

If FHR not heard, see below **

LIQUOR (I/C/B/M)

Blood stained liquor / Antepartum bleeding

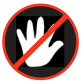

Observe for signs of shock *(page 6)*

Cause of bleeding?:

1. Abruptio placenta
2. Ruptured uterus
3. Placenta praevia
4. Vasa praevia
5. Other cause

! No PV until placenta praevia is ruled out by ultrasound, or in theatre with CS pack open and ready

Meconium

Can be a sign of fetal distress: Assess FHR and signs of obstruction *(page 4)*

! At delivery: IMMEDIATE suctioning of baby's nose and mouth *(before drying baby)*

! Pushing is the most dangerous time for the baby: FHR after every contraction & constant attendance

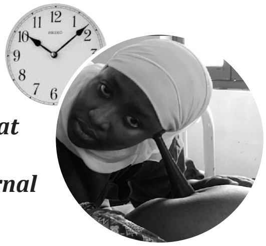

! Always ensure that it is FHR, not maternal pulse

* INTRAUTERINE RESUSCITATION

- Woman on left side
  (if no improvement, then right side)
- Stop oxytocin if administered
- Assess Pulse, BP, FHR, PV, Temp

** FHR NOT HEARD

Confirmation of absent FHR:

- Ask colleague to reassess FHR (with Dopptone) & perform ultrasound

Confirmed intrauterine fetal death:

Plan for vaginal birth:

- Induction/augmentation of labour
- Craniotomy *(if obstructed labour)*
- Observe for signs of infection and treat *(page 6)*
- Provide emotional support

! CS only as last option or if severe maternal compromise

Version 1.2

© *2015 The PartoMa Study, University of Copenhagen*

LATENT PHASE

Regular painful contractions & cervical ripening (softening, effacing, opening), cervix < 4 cm

Assess every 4 hrs. if admitted *(page 1-2)*

Signs of infection? *(PROM, UTI etc.)*

Need for pain relief?

! ***If prelabour rupture of membranes > 18 hrs., start antibiotics & plan for delivery*** *(induction/augmentation)*

Latent phase > 12 hrs.:

Poor progress in latent phase? This might need ARM & oxytocin augmentation **

! ***Prolonged latent phase is often confused with false labour where contractions cease after a while*** *(UTI?)*

FIRST STAGE OF ACTIVE PHASE OF LABOUR

Cervix ≥ 4 cm and regular painful contractions (how to assess PV and contractions, see page 1)

! ***No PV if placenta praevia is suspected*** *(page 3)*

Normal progress:

Routine assessments & Supportive care *(page 1-2)*

Next PV after 4 hrs.

Alert line

Early detection of poor progress:

Assess **5 Ps** *

Empty **Bladder**

If membranes intact: **ARM** *(If HIV positive, wait till action line is crossed or cervix ≥8cm)*

IV Normal Saline or Ringer's Lactate 250 mL/hr. *(If BP < 140/90)*

Ambulation

Next PV after 2 hrs.

..........4 hrs...........

Action line

Severe poor progress:

Assess **5 Ps** *

If not strong contractions 1 hr. after ARM & urination: **Oxytocin** **

PV every 2 hrs.

Decide on CS if:

- Progressive signs of obstruction *(no further dilatation & descent, moulding +++ and positive FHR)*
- No progress after 4 hrs. oxytocin
- Fetal or maternal compromise

SECOND STAGE OF ACTIVE PHASE OF LABOUR *Cervix fully dilated*

! EVEN IF POOR PROGRESS IN FIRST STAGE, PROGRESS IN SECOND STAGE STARTS IN GREEN ZONE

≤ 1 hr. and pushing ≤ 30 min.

Supportive / encouraging care

! ***Never leave the woman alone in second stage & monitor FHR closely*** *(page 3)*

1-2 hrs and/or pushing 30-60 min.

Exclude malposition

Consider augmentation: *(if presenting part not visible at vulva)*

- ARM & Oxytocin **
- IV Normal Saline or Ringer's Lactate 250 mL/hr *(if BP < 140/90)*

PV every 15-30 min.

> 2 hrs and/or pushing > 1 hr.

Vacuum extraction *(page 7) (if bony part of fetal head at or below ischial spines)*

CS as last resort

! ***Always stop Oxytocin infusion when deciding on CS***

* 5 Ps - *why poor progress of labour?*

Power:

If <4 strong contractions /10 min., augment: ARM, IV fluid, ambulation, oxytocin ** *(see specific instructions above)*

Passenger:

- Malposition/malpresentation? *(if yes, vaginal delivery possible?)*

Psyche:

- Encourage, reassure, reduce anxiety

Pass urine:

- Encourage spontaneous emptying every 2 hrs.
- Catheterize as last resort

Pelvis:

True CPD may be considered when failed trial of augmentation

** OXYTOCIN AUGMENTATION

Indication: Severe poor progress of labour and <4 strong contractions /10 min.

Start dose: Oxytocin 2.5 units in 500 ml Normal Saline or Ringer's Lactate at 10 drops/min.

Every 15 min.: Carefully count FHR, contractions, oxytocin drops per min.

Every 30 min.: Increase infusion rate by 5 drops/min. half hourly until 4-5 strong contractions/10 min. Maximum 60 drops/min. Maintain this rate until delivery.

! ***NEVER > 5 CONTRACTIONS per 10 MIN.*** *If hyperstimulation, stop oxytocin.*

© 2015 The PartoMa Study, University of Copenhagen

Version 1.2

HYPERTENSION & PRE-ECLAMPSIA / ECLAMPSIA

! THESE GUIDELINES ONLY INVOLVE TREATMENT DURING LABOUR AND DELIVERY

SBP ≥ 160 and/or DBP ≥ 110 *(mmHg)*

SEVERE Hypertension / Pre-eclampsia:

Medication (SLOWLY):
Anticonvulsant * & Antihypertensive **

Assess every 30 min. *(use the specific observation sheets)*:
- Pulse, BP, RR, Temp, FHR, GCS

Symptoms of organ failure * or Magnesium Sulphate toxicity *?**
Ask patient and assess lungs, urine output, urine dipstick, patellar reflexes

Strict fluid balance:
- Catheterize bladder (fluid intake & output, proteinuria)
- If urine output <30mL/hr.: IV Ringer's Lactate 1L in 8 hrs

Plan for delivery within 12 hrs. of admission (if at all possible, vaginal delivery is preferable)

Convulsions (eclampsia):

! *Treat as eclampsia until other diagnosis is confirmed*

1. Shout for help
2. Airways and breathing
3. Position on left side and protect from injuries
4. Insert IV lines
5. Start Magnesium Sulphate SLOWLY *
6. Oxygen by mask / nasally

Additional management as for severe pre-eclampsia

SBP 140-159 and/or DBP 90-109 *(mmHg)*

MILD-MODERATE Hypertension / Pre-eclampsia:

Ask & observe for symptoms of organ failure ***

Assess lungs, urine output, proteinuria, patellar reflexes

Reassess Pulse & BP every hr. FHR every 30 min.

! *For pre-eclampsia and eclampsia, specific observation & treatment sheets must be used in combination with the partogram*

SBP 100-139 and/or DBP 60-89 *(mmHg)*

NORMAL

BP every 4 hrs.
(for other routine assessments, see page 1)

* ANTICONVULSANT

MAGNESIUM SULPHATE (50%):

Loading dose:
IV 4 g in 250 mL Normal Saline **SLOWLY** over 15 min.
IM 5 g + 1 mL 2% Lignocaine in each buttock

Maintenance dose:
IM 5g + 1mL 2% Lignocaine every 4 hrs, alternate buttocks

Check for toxicity and DO NOT repeat dose if:
RR < 16/min.
Urinary output < 30 ml/hr.
Patellar reflexes diminished or absent

Antidote:
IV Calcium Gluconate 1 gram (10mL in 10% solution) over 10 min.

Duration:
Continue maintenance dose for 24 hrs. after delivery or last convulsion, whichever occurs last

** ANTIHYPERTENSIVE

HYDRALAZINE:
IV 5 mg **SLOWLY** over 10 min.
Repeat 5mg every 20 min. until SBP < 160 mmHg

! ***DBP should not fall below 90 mmHg***
! ***Observe closely:*** *Hydralazine may cause maternal hypotension and fetal distress*

*** DIAGNOSING PRE-ECLAMPSIA

MILD-MODERATE pre-eclampsia:
Hypertension on two consecutive readings AND
Proteinuria ≥ ++

SEVERE pre-eclampsia:
Pre-eclampsia **AND**
Severe hypertension **OR**
Symptoms of organ failure:
- Headache *(persistent & severe)*
- Blurred vision
- Upper abdominal pain *(persistent)*
- Decreased urine production *(< 30 ml/hr)*
- Breathlessness *(pulmonary oedema)*

Version 1.2

© 2015 The PartoMa Study, University of Copenhagen

LOW BLOOD PRESSURE (BP) OR LOW/HIGH PULSE

**SBP < 100 (mmHg)**<br><br>● **or Pulse < 60 bpm or Pulse > 110 bpm** *(also see below)*	**Immediate danger signs?:**<br>- **SBP < 90** *(SHOCK)*<br>- **Unconscious** *(if convulsions, see page 5)*<br>- **Cardiac arrest** *(START CARDIAC MASSAGE & VENTILATION)*<br><br>**Elevate legs**<br>**Vital signs every 15 min.:**<br>Pulse, BP, RR, temp, PV, blood loss, FHR, urine output<br>**Oxygen** *(by mask / nasal cannulae)*<br>**Collect blood**<br>Haemoglobin, blood group, cross-match and clotting-test<br>**Insert IV lines** *(wide bore cannula)*:<br>IV Normal Saline or Ringer's Lactate 2L in 20-40 min.<br>**Catheterize bladder** *(fluid intake and output)*<br><br>**Determine & manage cause:**<br>1. bleeding 4. cardiac<br>2. sepsis 5. other<br>3. trauma

HIGH PULSE (P)

● **Pulse > 100 OR FHR > 160**	**Consider maternal infection, dehydration or bleeding?**<br>- Pulse, BP, RR, Temp, FHR every 15-30 min.<br>- Uterine pain?<br>- PV *(blood loss, foulsmelling vaginal discharge)*<br>- Sufficient fluid intake?<br>**If P > 110:** SUSPECT SHOCK *(see above)*

HIGH TEMPERATURE (Temp)

**Temp ≥ 38°C = FEVER**	**Antibiotics until delivery:**<br>For example *(if not allergic)*:<br>Ampicillin 2g IV /6 hours AND Gentamicin 5 mg/kg IV /24 hrs.<br>**Tablet Paracetamol** 1 gram every 6 hrs.<br>**Consider diagnosis & order relevant laboratory tests:**<br>UTI, Chorioamnionitis, Malaria, Sepsis<br>*(remember to adjust treatment accordingly)*<br>**Plan for delivery within 12 hrs.**<br>*(If latent phase, augment labour, page 4)*<br>**Measure Pulse, BP, RR, FHR every 15-30 min. Measure Temp hourly**

© 2015 The PartoMa Study, University of Copenhagen

EMERGENCY CS - a few notes

Contraindications for CS:
If the woman is medically unstable (e.g. severe hypertension), it is recommended that the maternal condition is stabilized first, and delivery considered only for obstetric indications.

Maximum time from deciding on emergency CS to delivery:

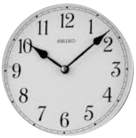

If fetal or maternal compromise: 30 minutes
(E.g. fetal distress, cord prolapse with pulsating cord, severe antepartum haemorrhage, maternal medical condition)

If no maternal or fetal compromise, but early delivery is needed: 75 minutes
(E.g. poor progress in active labour, 2 times previous lower segment CS, placenta praevia)

VACUUM EXTRACTION

This section is reproduced with permission from ALSO guidelines pocket booklet, issue of Continuing Medical Education.
Copyright: © American Academy of Family Physicians. All Rights Reserved.

Most important indications:
Fetal distress *(page 3)*
Poor progress in second stage of labour *(page 4)*

Required beforehand:
1. Cervix fully dilated
2. Cephalic presentation, membranes ruptured
2. Fetal head at or below ischial spines *(level 1/5 or 0/5)*
3. Gestational age 34 weeks or more
4. Birth attendant trained in vacuum extraction

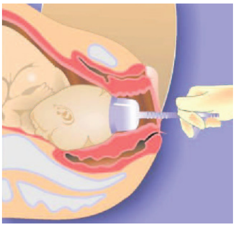

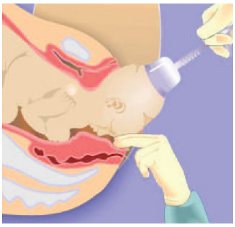

The A-J approach to vacuum extraction:

A **A**sk for help
Address the patient
(inform that you need patient to cooperate and keep pushing when there is contraction)
Abdominal Palpation *(descent of head)*

B **B**ladder empty?

C **C**ervix must be fully dilated
Contractions are needed *(oxytocin needed? Page 4)*

D **D**etermine position of the head
(locate the posterior triangular fontanel)

E **E**quipment ready?
(delivery tray, towels, neonatal resuscitator, vacuum extractor)

F **F**lexion point must be located
(place the edge of the cup at the tip of the posterior triangular fontanel)
Feel for vaginal tissue between cup and fetal skull to avoid perineal trauma
(before and after applying suction)

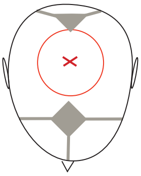

G **G**entle, steady traction with no rocking during contractions
(first contractions downward traction, during following contractions more upward)

H **H**ALT traction between contractions
HALT and abandon if 3 pop-offs
HALT if 3 pulls with no progress
HALT after 20 min. of use (if delivery not achieved)

I **I**ntact perineum! When head is delivered, protect perineum with one hand
(incision is only rarely needed)

J When the **J**aw is reachable, release vacuum and remove cup

! If the procedure is not possible or fails, CS should be performed immediately

! Compared to spontaneous vaginal birth, vacuum extraction has increased risk of perineal trauma and minor trauma to the head of the baby

Guidelines development team:
Nanna Maaløe, Tarek Meguid, Birgitte Bruun Nielsen, Jos van Roosmalen

Internal review:
The guidelines were reviewed twice at Mnazi Mmoja Hospital; first by six nurse-midwives and doctors, and secondly by a 4 weeks pilot testing and evaluation by 32 birth attendants.

External peer-review:
The PartoMa guidelines are peer-reviewed by seven international experts specialized in midwifery/obstetrics.

Continual adjustments to reach reality in the best possible way:
Suggestions for improvements are always welcome. Thank you to the entire group of birth attendants at Mnazi Mmoja Hospital who have helped adjusting the guidelines during the first year.

More information on the PartoMa study:
publichealth.ku.dk/sections/global/project/partoma/

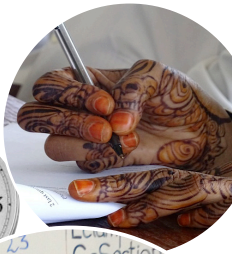

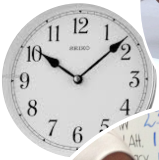

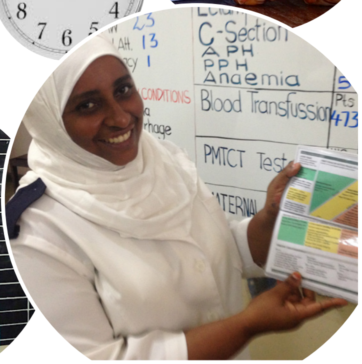

© 2015 The PartoMa Study, University of Copenhagen. All Rights Reserved. Requests for permission to reproduce or translate should be addressed by email to: techtrans@adm.ku.dk.

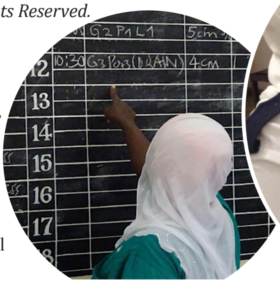

All reasonable precautions have been taken by the PartoMa study team to verify the information contained in this publication, and both text and graphical presentations are internationally peer-reviewed. The PartoMa guidelines are primarily developed to guide the health providers to the best possible care in the low resourced referral hospital of Zanzibar, Mnazi Mmoja Hospital. The material is being distributed without warranty of any kind, and the responsibility for the interpretation and use of the material lies with the reader.
