# Supplementary material for: Labour management guidelines for a Tanzanian referral hospital: The participatory development process and birth attendants’ perceptions
Source: BMC Pregnancy Childbirth. 2017 Jun 7;17:175. doi: 10.1186/s12884-017-1360-2 (PMC5463375; doi:10.1186/s12884-017-1360-2)
Supplement: Supplementary file 3 — A systematic literature search: Labour and delivery guidelines for African low-income settings. (PDF 401 kb) [file 12884_2017_1360_MOESM3_ESM.pdf]

A systematic literature search: Intrapartum guidelines for African hospitals

---

Summary: By a systematic search in PubMed combining search terms related to clinical guidelines, intrapartum care, and low resource settings, with specific focus on Africa (1st January 2000 – 6th July 2016, English language), we identified 43 published studies on guidelines development/modification, use, or effectiveness. Four studies concerned development/modification of guidelines, and eight evaluated guidelines implementation (one study was included in both of these categories). The remaining 32 publications compared actual clinical practice with expected best quality of care as recommended by well-established international evidence-based guidelines. All of the latter 32 analyses concluded clinical practice to be alarmingly suboptimal when compared to international guidelines; 15 of these, representing 11 sub-Saharan African countries, specifically called for simpler and more achievable guidance. While international guidelines production for intrapartum care appears to have increased rapidly since 2000, published literature suggests that it has only in few instances been matched with reviews of local modifications, use, and impact at the targeted low resource facilities.

Objective: To quantify the number and summarize the content of published studies on intrapartum guidelines for low income settings, with specific focus on Africa.

Eligibility criteria:

Topic: Intrapartum clinical guidelines, defined as guidelines for the period from the onset of labour to the end of the third stage of labour.

Types: All published studies, including reviews, concerning:

1. Development/modifications/adaptations of guidelines
2. Implementation of guidelines
3. Evaluation of guidelines use and effect, including perceptions among staff, knowledge/skills changes, and resulting changes in practice and outcome

Exclusion criteria: Articles solely reporting on drugs regimens or single procedures.

Settings: The main focus of the search was African low resource hospitals. However, when found by the search, relevant studies from comparable low resource settings in other parts of the world were included as well.

Time interval: 1st January 2000 – 5th July 2016

Language: English

Database: Pubmed

Search strategy: **Main search:**

Search parts	Guidelines	Intrapartum	Low resource settings
**Mesh terms**	Practice Guidelines as Topic	Labour, obstetric<br>Parturition	Poverty<br>Developing countries<br>Africa
**Free text**	Guideline(s)<br>“Best practice(s)”<br>Standard(s)[Title]<br>Instruction(s)[Title]	Intrapartum<br>Birth<br>Childbirth<br>Parturition	“Low income”<br>Africa<br>“Developing countries”<br>“Developing country”

(((((((Instruction[Title]) OR Standard*[Title])) OR (Guidelines OR "Best practices" OR "Best practice")) OR "Practice Guidelines as Topic"[Mesh])) AND (((("Labor, Obstetric"[Mesh]) OR "Parturition"[Mesh])) OR (Parturition OR Intrapartum OR Birth OR Childbirth))) AND ((((("Poverty"[Mesh]) OR "Developing Countries"[Mesh]) OR "Africa"[Mesh])) OR ("Low income" OR Africa OR "Developing countries" OR "Developing country"))*

Supplementary search:

A focused search was conducted on the World Health Organization’s guidelines for managing complications in pregnancy and childbirth:

Search parts	World health organization	The IMPAC guidelines
**Mesh terms**	World Health Organization	
**Free text**	“World Health Organization”<br>WHO	"managing complications in pregnancy and childbirth"<br>"integrated management of pregnancy and childbirth"<br>IMPAC

(((("World Health Organization"[Mesh]) OR "World Health Organization") OR WHO)) AND (((("managing complications in pregnancy and childbirth")) OR ("integrated management of pregnancy and childbirth")) OR IMPAC)

Study selection: The main broad search resulted in 405 titles. After a scan of abstracts and full text articles where relevant, 42 publications followed the eligibility criteria and were included. In the vast majority of excluded publications, the term guidelines or its synonyms was e.g. used in methodological sections or in debates/discussions without the eligibility criteria being met. In addition, some publications concerned antenatal or neonatal care, or child health, which lay outside the scope of this review.

The supplementary search resulted in 32 titles, of which one additional quality assurance study was included, resulting in a total of 43 included publications.

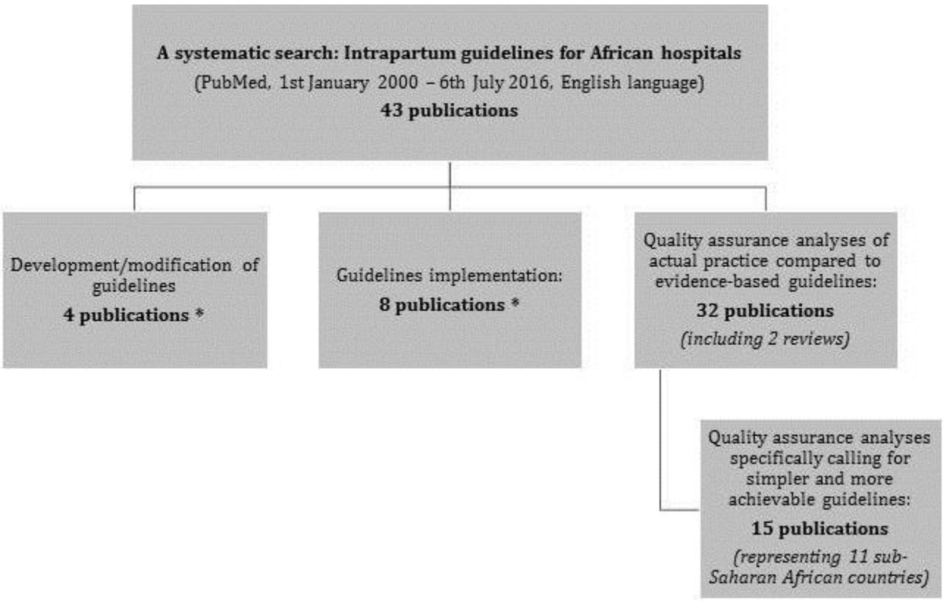

Figure S1 A systematic literature search of intrapartum guidelines for African hospitals; synthesis of the results. *One study is included in both categories.

Synthesis of results (Figure S1):

Four publications described the development or modification process of guidelines. In two of the publications, Kongnyuy et al. described participatory national approaches for development of standards for obstructed labour and women friendly intrahospital care in Malawi.[1,2] They concluded that while international guidelines were traditionally based on expert consensus, it was beneficial to include the actual end users in the processes. As part of a package to improve emergency obstetric care in two rural districts of Mali, Otchere et al. briefly described that they in collaboration with hospital partners, the Ministry of Health, and others developed clinical protocols for the targeted facilities.[3] Finally, Ameh et al. described the development process, including feedback from Nigerian birth attendants, of a severe pre-eclampsia/eclampsia monitoring and treatment sheet (the LIVKAN chart), which includes guidelines.[4]

Eight publications concerned evaluation of implemented guidelines. A study from a community hospital in Senegal implemented emergency obstetric guidelines by criterion-based audit and feedback and evaluated the effect on clinical practice.[5] In two publications, the QUALMAT study team presented multicenter evaluation of a computer-assisted clinical decision support system for antenatal and delivery care in sub-Saharan Africa, which includeded guidelines.[6,7] They explored both staff's

perceptions and the effect on maternal and perinatal outcome. A one-center study from India evaluated effects of the WHO Safe Birth Checklist, which included guidelines, on clinical practice.[8] Two single-hospital studies from tertiary facilities in Tanzania and Pakistan evaluated the effect of locally developed guidelines for eclampsia and postpartum haemorrhage, respectively, on clinical practice and outcome.[9,10] One study from Malawi evaluated national guidelines regarding the obstructed labour guidelines described above.[11] Finally, in the LIVKAN publication described above, birth attendants' immediate perceptions to the new chart was included.[4] All publications concluded their guidelines to show promising effects.

The remaining 32 publications reported on quality assurance studies of intrapartum care compared to well-established international evidence-based guidelines.[12–43] They presented findings from one or more low resource facilities, and quantitative, qualitative, and mixed methods approaches were applied. All studies concluded intrapartum care to be suboptimal when compared to evidence-based best practice. Of the studies, 15 (47%), representing 11 sub-Saharan African countries, specifically stressed the need for useful guidelines in their facilities.[12–26] Others stressed the need for strengthening supplies, staff numbers, and training in order to follow established international guidelines. Two of the publications presented reviews of criterion-based audits concerning intrapartum care and reached similar conclusions.[42,43]

Conclusion: Multiple quality assurance studies from low resource settings conclude intrapartum care to be suboptimal when compared to well-established recommendations of best practice, and the need for locally useful guidelines is repeatedly stressed. However, while international guidelines production for intrapartum care appears to have increased rapidly since 2000, it has not been acceptably matched with reviews of actual use and impact at the targeted low resource facilities.

References:

1. Kongnyuy EJ, Mlava G, van den Broek N. Establishing standards for obstructed labour in a low-income country. Rural Remote Health [Internet]. [cited 2016 Jun 8];8(3):1022. Available from: http://www.ncbi.nlm.nih.gov/pubmed/18690763

2. Kongnyuy EJ, van den Broek N. Criteria for clinical audit of women friendly care and providers' perception in Malawi. BMC Pregnancy Childbirth [Internet]. 2008 [cited 2016 Jul 7];8:28. Available from: http://www.ncbi.nlm.nih.gov/pubmed/18647388

3. Otchere SA, Kayo A. The challenges of improving emergency obstetric care in two rural districts in Mali. Int J Gynecol Obstet. 2007;99(2):173–82.

4. Ameh CA, Ekechi CI, Tukur J. Monitoring severe pre-eclampsia and eclampsia treatment in resource poor countries: skilled birth attendant perception of a new treatment and monitoring chart (LIVKAN chart). Matern Child Health J. 2012;16(5):941–6.

5. Dumont A, Gaye A, Mahé P, Bouvier-Colle M-H. Emergency obstetric care in developing countries: impact of guidelines implementation in a community hospital in Senegal. BJOG [Internet]. 2005 Sep 1 [cited 2017 Mar 21];112(9):1264–9. Available from: http://doi.wiley.com/10.1111/j.1471-0528.2005.00604.x

6. Sukums F, Mensah N, Mpembeni R, Massawe S, Duysburgh E, Williams A, et al. Promising adoption of an electronic clinical decision support system for antenatal and intrapartum care in rural primary healthcare facilities in sub-Saharan Africa: The QUALMAT experience. Int J Med Inform [Internet]. 2015 Sep [cited 2016 Jun 8];84(9):647–57. Available from: http://www.ncbi.nlm.nih.gov/pubmed/26073076

7. Dalaba MA, Akweongo P, Williams J, Saronga HP, Tonchev P, Sauerborn R, et al. Costs associated with implementation of computer-assisted clinical decision support system for antenatal and delivery care: case study of Kassena-Nankana district of northern Ghana. PLoS One [Internet]. 2014 [cited 2016 Jul 7];9(9):e106416. Available from: http://www.ncbi.nlm.nih.gov/pubmed/25180831

8. Spector JM, Agrawal P, Kodkany B, Lipsitz S, Lashoher A, Dziekan G, et al. Improving quality of care for maternal and newborn health: prospective pilot study of the WHO safe childbirth checklist program. PLoS One [Internet]. 2012 [cited 2016 Jun 8];7(5):e35151. Available from: http://www.ncbi.nlm.nih.gov/pubmed/22615733

9. Kidanto HL, Wangwe P, Kilewo CD, Nystrom L, Lindmark G. Improved quality of management of eclampsia patients through criteria based audit at Muhimbili National Hospital, Dar es Salaam, Tanzania. Bridging the quality gap. BMC Pregnancy Childbirth [Internet]. 2012 [cited 2016 Jul 7];12:134. Available from: http://www.ncbi.nlm.nih.gov/pubmed/23170817

10. Sheikh L, Najmi N, Khalid U, Saleem T. Evaluation of compliance and outcomes of a management protocol for massive postpartum hemorrhage at a tertiary care hospital in Pakistan. BMC Pregnancy Childbirth [Internet]. 2011 [cited 2016 Jul 7];11:28. Available from: http://www.ncbi.nlm.nih.gov/pubmed/21489279

11. Kongnyuy EJ, Mlava G, van den Broek N. A criterion based audit of the management of obstructed labour in Malawi. Arch Gynecol Obstet [Internet]. 2009 May [cited 2016 Jul 7];279(5):649–54. Available from: http://www.ncbi.nlm.nih.gov/pubmed/18779971

12. M'Rithaa DKM, Fawcus S, Korpela M, De la Harpe R. The expected and actual communication of health care workers during the management of intrapartum: An interpretive multiple case study. African J Prim Heal care Fam Med [Internet]. 2015 [cited 2016 Jul 7];7(1):911. Available from: http://www.ncbi.nlm.nih.gov/pubmed/26842518

13. Bartlett L, Cantor D, Lynam P, Kaur G, Rawlins B, Ricca J, et al. Facility-based active management of the third stage of labour: assessment of quality in six countries in sub-Saharan Africa. Bull World Health Organ [Internet]. 2015 Nov 1 [cited 2016 Jul 7];93(11):759–67. Available from: http://www.ncbi.nlm.nih.gov/pubmed/26549903

14. Oguntunde O, Charyeva Z, Cannon M, Sambisa W, Orobaton N, Kabo IA, et al. Factors influencing the use of magnesium sulphate in pre-eclampsia/eclampsia management in health facilities in

Northern Nigeria: a mixed methods study. BMC Pregnancy Childbirth [Internet]. 2015 [cited 2016 Jul 7];15:130. Available from: http://www.ncbi.nlm.nih.gov/pubmed/26037906

15. Madzimbamuto FD, Ray SC, Mogobe KD, Ramogola-Masire D, Phillips R, Haverkamp M, et al. A root-cause analysis of maternal deaths in Botswana: towards developing a culture of patient safety and quality improvement. BMC Pregnancy Childbirth. 2014;14:231.

16. Schack SM, Elyas A, Brew G, Pettersson KO. Experiencing challenges when implementing active management of third stage of labor (AMTSL): a qualitative study with midwives in Accra, Ghana. BMC Pregnancy Childbirth [Internet]. 2014 [cited 2016 Jul 7];14:193. Available from: http://www.ncbi.nlm.nih.gov/pubmed/24903893

17. Chuma C, Kihunrwa A, Matovelo D, Mahendeka M. Labour management and Obstetric outcomes among pregnant women admitted in latent phase compared to active phase of labour at Bugando Medical Centre in Tanzania. BMC Pregnancy Childbirth. 2014;14:68.

18. Yisma E, Dessalegn B, Astatkie A, Fesseha N. Completion of the modified World Health Organization (WHO) partograph during labour in public health institutions of Addis Ababa, Ethiopia. Reprod Health [Internet]. 2013 [cited 2016 Jul 7];10:23. Available from: http://www.ncbi.nlm.nih.gov/pubmed/23597239

19. Gans-Lartey F, O'Brien BA, Gyekye FO, Schopflocher D. The relationship between the use of the partograph and birth outcomes at Korle-Bu teaching hospital. Midwifery. 2013;29(5):461–7.

20. Spangler SA, Spangler S, Bloom S, Organization WH, Organization WH, Graham W, et al. Assessing skilled birth attendants and emergency obstetric care in rural Tanzania: the inadequacy of using global standards and indicators to measure local realities. Reprod Health Matters. 2012 Jun;20(39):133–41.

21. Maaløe N, Bygbjerg IC, Onesmo R, Secher NJ, Sorensen BL. Disclosing doubtful indications for emergency cesarean sections in rural hospitals in Tanzania: A retrospective criterion-based audit. Acta Obstet Gynecol Scand. 2012;91(9):1069–76.

22. Maaløe N, Sorensen BL, Onesmo R, Secher NJ, Bygbjerg IC. Prolonged labour as indication for emergency caesarean section: A quality assurance analysis by criterion-based audit at two Tanzanian rural hospitals. Vol. 119, BJOG. 2012. p. 605–13.

23. Ogwang S, Karyabakabo Z, Rutebemberwa E. Assessment of partogram use during labour in Rujumbura Health Sub District, Rukungiri District, Uganda. Afr Health Sci [Internet]. 2009 Aug 1 [cited 2016 Jul 7];9 Suppl 1:S27–34. Available from: http://www.ncbi.nlm.nih.gov/pubmed/20589158

24. Oladapo OT, Akinola OI, Fawole AO, Adeyemi AS, Adegbola O, Loto OM, et al. Active management of third stage of labor: evidence versus practice. Acta Obstet Gynecol Scand [Internet]. 2009 [cited 2016 Jul 7];88(11):1252–60. Available from: http://www.ncbi.nlm.nih.gov/pubmed/19824866

25. Kidanto HL, Mogren I, Massawe SN, Lindmark G, Nystrom L. Criteria-based audit on management of eclampsia patients at a tertiary hospital in Dar es Salaam, Tanzania. BMC Pregnancy Childbirth [Internet]. 2009 [cited 2016 Jul 7];9:13. Available from: http://www.ncbi.nlm.nih.gov/pubmed/19323846

26. Kaye D. Quality of midwifery care in Soroti district, Uganda. East Afr Med J [Internet]. 2000 Oct [cited 2016 Jul 7];77(10):558–61. Available from: http://www.ncbi.nlm.nih.gov/pubmed/12862125

27. Dettrick Z, Gouda HN, Hodge A, Jimenez-Soto E. Measuring Quality of Maternal and Newborn Care in Developing Countries Using Demographic and Health Surveys. PLoS One [Internet]. 2016 [cited 2016 Jul 7];11(6):e0157110. Available from: http://www.ncbi.nlm.nih.gov/pubmed/27362354

28. Austin A, Gulema H, Belizan M, Colaci DS, Kendall T, Tebeka M, et al. Barriers to providing quality emergency obstetric care in Addis Ababa, Ethiopia: Healthcare providers' perspectives on training, referrals and supervision, a mixed methods study. BMC Pregnancy Childbirth [Internet]. 2015 [cited 2016 Jul 7];15:74. Available from: http://www.ncbi.nlm.nih.gov/pubmed/25885336

29. Owens L, Semrau K, Mbewe R, Musokotwane K, Grogan C, Maine D, et al. The state of routine and emergency obstetric and neonatal care in Southern Province, Zambia. Int J Gynecol Obstet. 2015;128(1):53–7.

30. Aminu M, Utz B, Halim A, van den Broek N. Reasons for performing a caesarean section in public hospitals in rural Bangladesh. BMC Pregnancy Childbirth [Internet]. 2014 [cited 2016 Jul 7];14:130. Available from: http://www.ncbi.nlm.nih.gov/pubmed/24708738

31. Yisma E, Dessalegn B, Astatkie A, Fesseha N. Knowledge and utilization of partograph among obstetric care givers in public health institutions of Addis Ababa, Ethiopia. BMC Pregnancy Childbirth [Internet]. 2013 [cited 2016 Jul 7];13:17. Available from: http://www.ncbi.nlm.nih.gov/pubmed/23331626

32. Vivio D, Fullerton JT, Forman R, Mbewe RK, Musumali M, Chewe PM. Integration of the Practice of Active Management of the Third Stage of Labor Within Training and Service Implementation Programming in Zambia. J Midwifery Womens Health. 2010;55(5):447–54.

33. Getaneh W, Kumbi S. Use of magnesium sulfate in pre-eclampsia and eclampsia in teaching hospitals in Addis Ababa: a practice audit. Ethiop Med J [Internet]. 2010 Apr [cited 2016 Jul 7];48(2):157–64. Available from: http://www.ncbi.nlm.nih.gov/pubmed/20608019

34. Ijadunola KT, Ijadunola MY, Esimai OA, Abiona TC. New paradigm old thinking: The case for emergency obstetric care in the prevention of maternal mortality in Nigeria. BMC Womens Health [Internet]. 2010 [cited 2016 Jul 7];10:6. Available from: http://www.ncbi.nlm.nih.gov/pubmed/20163719

35. Oladapo OT, Fawole AO, Loto OM, Adegbola O, Akinola OI, Alao MO, et al. Active management of third stage of labour: a survey of providers' knowledge in southwest Nigeria. Arch Gynecol Obstet [Internet]. 2009 Dec [cited 2016 Jul 7];280(6):945–52. Available from: http://www.ncbi.nlm.nih.gov/pubmed/19306012

36. SEA-ORCHID Study Group, Laopaiboon M, Lumbiganon P, McDonald SJ, Henderson-Smart DJ, Green S, et al. Use of evidence-based practices in pregnancy and childbirth: South East Asia Optimising Reproductive and Child Health in Developing Countries project. PLoS One [Internet]. 2008 [cited 2016 Jul 7];3(7):e2646. Available from: http://www.ncbi.nlm.nih.gov/pubmed/18612381

37. Harvey SA, Blandón YCW, McCaw-Binns A, Sandino I, Urbina L, Rodríguez C, et al. Are skilled birth attendants really skilled? A measurement method, some disturbing results and a potential way forward. Bull World Health Organ [Internet]. 2007 Oct [cited 2016 Jul 7];85(10):783–90. Available from: http://www.ncbi.nlm.nih.gov/pubmed/18038060

38. Khalil K, Elnoury A, Cherine M, Sholkamy H, Hassanein N, Mohsen L, et al. Hospital practice versus evidence-based obstetrics: categorizing practices for normal birth in an Egyptian teaching hospital. Birth. 2005 Dec;32(4):283–90.

39. Harvey SA, Ayabaca P, Bucagu M, Djibrina S, Edson WN, Gbangbade S, et al. Skilled birth attendant competence: an initial assessment in four countries, and implications for the Safe Motherhood movement. Int J Gynecol Obstet. 2004;87(2):203–10.

40. Khalil K, Cherine M, Elnoury A, Sholkamy H, Breebaart M, Hassanein N. Labor augmentation in an Egyptian teaching hospital. Int J Gynaecol Obstet [Internet]. 2004 Apr [cited 2016 Jul 7];85(1):74–80. Available from: http://www.ncbi.nlm.nih.gov/pubmed/15050479

41. Ith P, Dawson A, Homer C. Quality of maternity care practices of skilled birth attendants in Cambodia. Int J Evid Based Healthc [Internet]. 2012 Mar [cited 2016 Jul 7];10(1):60–7. Available from: http://www.ncbi.nlm.nih.gov/pubmed/22405417

42. Kerber KJ, Mathai M, Lewis G, Flenady V, Erwich JJHM, Segun T, et al. Counting every stillbirth and neonatal death through mortality audit to improve quality of care for every pregnant woman and her baby. BMC Pregnancy Childbirth. 2015;15 Suppl 2:S9.

43. Merali HS, Lipsitz S, Hevelone N, Gawande AA, Lashoher A, Agrawal P, et al. Audit-identified avoidable factors in maternal and perinatal deaths in low resource settings: a systematic review. BMC Pregnancy Childbirth [Internet]. 2014 [cited 2016 Jul 7];14:280. Available from: http://www.ncbi.nlm.nih.gov/pubmed/25129069
